# EVALUATION OF THE RELATIONSHIP BETWEEN ANDROGENETIC ALOPECIA AND DEMODEX INFESTATION

**DOI:** 10.4103/0019-5154.41647

**Published:** 2008

**Authors:** Javidi Zari, Fata Abdolmajid, Maleki Masood, Mashayekhi Vahid, Nahidi Yalda

**Affiliations:** *From Mashhad University of Medical Sciences, St., Ghoraishy Building, P.O. Box: 91375-345, Mashhad, Iran*

**Keywords:** *Alopecia androgenetic alopecia*, *demodex*

## Abstract

**Introduction:**

Androgenetic alopecia (AGA) is one of the most common dermatologic disorders with a multifactorial etiology. Inflammatory activators such as Demodex infestation may play a role in the pathogenesis of some cases of androgenetic alopecia that do not respond to common treatments such as minoxidil and finasteride. The goal of this study is to evaluate the relationship between Demodex infestation and AGA.

**Materials and Methods:**

In this case-control study, 41 patients with AGA referred to the Dermatology Clinic of Imam Reza Hospital and 33 healthy individuals were evaluated as control. All of them were between 20 and 40 years old men. In order to identify Demodex infestation they were referred to the Parasitology laboratory.

**Results:**

Demodex was detected in 19.5% of patients and 15.2% of controls; therefore, there was no significant relationship between them statistically (*P* = 0.0787). Most of patients (85.4%) had greasy hair. The most common pattern of baldness was II degree in Hamilton scale.

**Conclusion:**

There is no relation between AGA and Demodex.

## Introduction

Androgenetic alopecia (AGA) is one of the most common dermatologic disorders, which is concerned more recently.

In 1951, Hamilton showed that 50% of men and 40% of women demonstrated AGA by the age of 50.^1^ In a study conducted in 1993, 40% males and 30% females have showed AGA.^2^ At present, AGA is considered to be an alteration of hair growth and/or the premature aging of the pilosebaceous unit with a multifactorial and even polygenic etiology.^3^

Various hypotheses have been put forward on the AGA causes, out of which none of them has a 100% role in it. According to our knowledge, the interfering factors are hormones, receptors, heredity and microbial flora.

A study on twins shows that predisposition, age of onset, pattern and progression rate of AGA are reflected by genetics.^4^,^5^ Treatment with antihypertensitive agents such as minoxidil or modulators of androgen metabolism such as finasteride are barely effective on 30%; this implies that other pathways may be envisioned.^3^,^6^ Nowadays, inflammation has been considered to be involved in pathogenesis of this disorder.^7^,^8^ In several studies on hair follicles taken from subjects with AGA a moderate perifollicular lymphohistiocytic inflammation in 30% of AGA and 15% of control cases is observed.^9^–^11^

Only 55% of male-pattern AGA patients with microinflammation had hair regrowth in response to minoxidil treatment, which was less than the 77% of patients with no signs of inflammation. This suggested that perifollicular inflammation may account for some cases of male-pattern AGA that do not respond to minoxidil to a certain extent.^12^ Inflammation is probably effective by altering the local hormone metabolism in initiating AGA.^6^ Vast investigations have been carried out on the probable role of *Demodex follicularum* as an inflammation-inducing agent. The role of demodex in AGA has been evaluated to be direct in some studies and indirect in others.^13^ By infiltrating the sebaceous gland of hair follicles, demodex causes an immune response and inflammation of surrounding tissue. Through long-term invasion, the parasite exhausts the hair bulb and shifts the hair cycle from anagen to telogen.^14^

The Nioxin Research Center Laboratories in Atlanta conducted the initial study on 54 subjects and showed that 53 out of 54 showed increased demodex in their alopecic scalps.^6^

In two studies by Dr. Vollmer and Dr. Fabin Forton, the role of demodex as an inflammation-inducing agent has been presented.^8^,^13^

This study was carried out, considering the abovementioned facts to evaluate the probable role of demodex infestation in androgenetic alopecia.

## Materials and Methods

This investigation was a case-control study conducted for 8 month, i.e., from Oct 23, 2003 to June 21, 2004. All the subjects were males aging between 20 and 40 years of age. The case group consisted of 41 males referred to Dermatology Clinic of Imam Reza Hospital with hair-loss presentation without any other disease; male pattern baldness (MPB) was confirmed by the dermatologist. The control group consisted of 33 medical and dentistry male students who were not suffering from hair loss and any other diseases. Age, hair-washing intervals and influence of other diseases were considered to be matched in the two groups.

To collect the necessary data, a questionnaire containing demographic and clinical information was used. The obtained data involved the AGA family history, hair type (greasy, normal and dry) and hair-washing intervals, severity of infestation with demodex, alopecia pattern and previous treatment records. Subjects who had treatment less than a month prior to this study were not admitted in order to omit any possible effects of previous treatment on infestation.

In order to examine the infestation severity with demodex, subjects were referred to the Parasitology Laboratory. Results were reported as 0 to 4+ on the basis of quality. Measuring errors was crossed out since the matched place and examination method on all case and control subjects. Ultimately, 41 patients and 33 control individuals were checked out; information was extracted from questionnaires and analyzed by using SPSS software (version 11.5).

## Results

The average age of the case group was 26.37 ± 2.28 years and the control group was 23.94 ± 2.18 years, which does not represent a significant difference.

About 80.5% of the case group and 51.5% of the control group had greasy hair and the difference of greasiness of hairs between these two groups was significant (*P* = 0.007). No significant statistical differences were observed between the hair-washing interval of the case group and control groups.

On examining the hair-loss pattern, 39% of the case group had Hamilton grade II. Among those with positive family history of AGA, 42.2% had grade II, and in those without family history, 50% had grade IV. No significant relation was observed between family history and Hamilton grade.

In the case group, 56.7% of the patients were not previously treated, and among those with a treatment record with more than 1 month prior to our study, the highest percent was with minoxidil (24.3%) and ketoconazol shampoo (14.6%). About 19.5% of case and 15.2% of control groups were infested with demodex, and the difference between the two groups was not significant (with 95% accuracy *P* = 0.787, Cl = 0.216-2.509) (Table 1).

**Table 1:** Comparison between the case group and thecontrol group with AGA referred to dermatology clinicto detect demodex infestation from October 23, 2003 to June 21, 2004

Group infested with demode	Case		Control	
		
	No.	%	No.	%
0	33	80.5	28	84.8
1+	5	12.2	3	9.1
2+	2	49	2	6.1
3+	1	2.4	0	0
4+	0	0	0	0
Result	*P* = 0.787		X2 = /0.057	

We excluded any previous treatment effects on demodex infestation by not admitting the patients with treatment less than one month prior to study as the inclusion criteria; however, in order to delete any possible effects of previous treatments, the degree of demodex infestation among cases not having previous treatment was compared with the control subjects. There was also no proven significant relation (*P* = 0.68) (Table 2).

**Table 2:** Comparison of demodex infestation severity among patients with AGA who are not receiving treatment with the control subjects referred to Dermatology Clinic of Imam Reza Hospital from Oct 23, 2003 to June 21 2004

Group infected with demodex	Case		Control	
		
	No.	%	No.	%
0	19	82.6	28	84.8
1+	2	8.7	3	9.1
3+	1	4.3	0	0
4+	0	0	0	0
Total	23	100	33	100

## Discussion

Androgenetic alopecia, which is by far the most common cause of hair loss, is a change in the hair-growth process or premature ageing of pilosebaceous unit with multifactorial etiology.^15^–^16^ It affects approximately 50% of men and 20-53% of women by the age of 50 years. Although it is a medically benign condition, it is a significant psychosocial issue for many patients. It has been accompanied with negative body image and compatibility disorder.^17^,^18^

Various factors are said to be effective in AGA pathogenesis, such as androgens, receptors, microbial flora, endogenous and exogenous stress and genetic imbalance. The fact that the success rate of treatment with either topical agents such as minoxidil or androgen metabolism modulators such as finasteride barely exceeds 30% implies that other pathways such as inflammation, 11-β; hydroxy steroid dehydrogenase(11-βHSD) activity or microorganism colonization may be involved in AGA pathogenesis.^3^

One of which to be most considered in AGA etiology is different inflammatory activators.^19^,^20^ Fifty percent of the scalp samples from patients with AGA had an inflammatory infiltrate of mononuclear cells and lymphocytes.^21^ In a study by Abell, 70% of case subjects and 30% of control subjects had inflammation and perifollicular fibrosis.^22^ In another study, 37% of AGA cases had a certain degree of inflammation and fibrosis.^9^ Whiting in a histopathologic study showed that there is a perifollicular lymphohistiocytic inflammation in 30% AGA cases and 10% control subjects, besides, in another study by Whiting *et al.*, only 55% males with AGA and micro-inflammation responded to minoxidil. This was less than the percentage of patients (77%) with no signs of inflammation. Hence, it is possible that perifollicular micro-inflammation may account for some cases of male pattern AGA that do not respond to minoxidil.^11^,^12^

Jaworsky presented that inflammation reaction in AGA is confined to the surrounding area of sebaceous glands and infandibulum and follicular infiltration with activated T cells results in the induced synthesis of collagen by dermal sheath fibroblasts, and ultimately, the replacement of hair follicle with fibrosis takes place.^12^,^23^ Therefore, the leading process to AGA is miniaturization and involvement of pilosebaceous units secondary to dermal sheath fibroplasias.^3^,^7^,^23^ On the other hand, inflammation can be involved in AGA pathogenesis by altering local hormone metabolism.^6^ Considering the abovementioned evidences, many investigations on AGA have focused on inflammation, and it can be a satisfactory opportunity to achieve new treatments.

Indirect role of demodex by inducing inflammation in the scalp skin in AGA pathogenesis has been suggested in many studies. Demodex mite is an obligatory human ectoparasite and it is resident in pilosebaceous units in which only two types of such units consist of demodex folicularum and brevis are known to be present in humans.^24^ The roles of this ectoparasite in disorders such as rosacea,^25^,^26^ acarica blepharitis,^27^,^28^ pityriasis follicularis, pustslar folliculitis, skin lesions of immunosuppressed patients^28^ and demodicidosis gravis are considered.^29^ Demodex mite has an immunoative lipase that is possibly responsible for the induction of inflammation^30^ or perhaps the prolonged invasion of hair follicle by demodex induces immune reaction, hair follicle exhaustion and ultimately the shift of the hair cycle from anagen phase to telogen.^31^

In a study on 388 hairs follicles from 24 skin samples, Demodex mite was found in 24% of inflamed and in 10% of noninflammed follicles, while 83% of follicles with demodex were inflamed. The investigations of Nioxin center in a study on 54 patients with AGA proved that 53 out of 54 cases had demodex in the scalp skin.^6^ In their study a study conducted by Larry *et al.* on 99 patients with racial differentiation, the following results were obtained:

Caucasian cases {40 (+), 32 (-) for demodex}, African-American cases {9(+), 9 (-)}, Hispanics {all 5 (+)}, and in Asian cases {all 4 (-)}. In total, 87.3% cases with AGA were positive for demodex. There is also relation between hair thinning and demodex infestation.^6^ Inflammatory responses due to demodex are said to be direct, indirect or even coincidental in different sources.

In our study to evaluate the probable relation between demodex infestation and AGA, infestation in the case group was 19.5% and in the control group was 15.2%; no significant difference was achieved (*P* = 0.787). Demodex mite has two species, from which *Demodex brevis* has a lower prevalence and wider distribution on the body, while face is most heavily infested by both the species. Males have higher rate of infestation than females, particularly with *Demodex brevis*.^32^ Therefore, various factors are responsible for demodex distribution on body. These two mite species differ in the type of involved follicle and prifollicular inflammation since area infested with *Demodex follicularum* is around the hair follicles and in the case of *Demodex brevis*, it is around the sebaceous gland secretary ducts. Inflammatory infiltrate in AGA is slightly lower than the skin surface corresponding to areas infestated with *Demodex follicularum*.^30^ Furthermore, body immune reactions towards these two species of demodex are different.^33^,^34^ Therefore, we should consider the demodex type while evaluating the role of demodex as well. Probably, the reason for finding no relation between AGA and demodex infestation in our study was our inability to determine the species of demodex in the cases due to lack of facilities. The method of sampling can also affect on results - as obtained in the following studies: Dr Larry's study, Nioscope (nonaggressive method that provides magnification and visualization of the hair follicle) and in a study by Dr. Volmer, scalp skin biopsy has been used, in which we take samples by scalp skin scraping and definitely aggressive methods such as biopsy can end up with more accurate results. Possibly, demodex density in scalp skin smear is not necessarily representing demodex around the hair follicles. The possibility of demodicidosis clinical signs is more in cases with special types of HLA and also in some HLA types that are resistant to demodecidosis,^30^ this explains the racial differences in Larry's study.^6^ The more likely explanation for the relation between demodex and AGA is that the sebaceous glands of alopecia-affected hair follicles become larger and more active, producing oils at a faster rate, under the influence of dihydrotestosterone (DHT), and hence, these follicles become a more suitable environment for demodex. In fact demodex infestation is considered to be secondary to AGA and not to its cause; this concept also explains the presence of greasy hair in our patients.^14^

## Conclusion

According to this study, we could not prove any relation between androgenetic alopecia and demodex infestation. On completion, we recommended more comprehensive studies on ecologic and immunologic characteristics of demodex species and a comparative review between the different methods for examining demodex infestation to be carried out. Furthermore, the effect of antidemodex treatments on AGA is another point that needs large-scale studies with greater feasibilities.
